# Evaluating Surgical Resection Extent and Adjuvant Therapy in the Management of Gliosarcoma

**DOI:** 10.3389/fonc.2020.00337

**Published:** 2020-03-11

**Authors:** Michael C. Jin, Elisa K. Liu, Siyu Shi, Iris C. Gibbs, Reena Thomas, Lawrence Recht, Scott G. Soltys, Erqi L. Pollom, Steven D. Chang, Melanie Hayden Gephart, Seema Nagpal, Gordon Li

**Affiliations:** ^1^Department of Neurosurgery, Stanford University Medical Center, Stanford, CA, United States; ^2^Department of Radiation Oncology, Stanford University Medical Center, Stanford, CA, United States; ^3^Department of Neurology and Neurological Sciences, Stanford University Medical Center, Stanford, CA, United States

**Keywords:** glioblastoma, gliosarcoma, temozolomide, adjuvant, brain tumor, neurosurgery

## Abstract

**Introduction:** Gliosarcomas are clinically aggressive tumors, histologically distinct from glioblastoma. Data regarding the impact of extent of resection and post-operative adjuvant therapy on gliosarcoma outcomes are limited.

**Methods:** Patients with histologically confirmed gliosarcoma diagnosed between 1999 and 2019 were identified. Clinical, molecular, and radiographic data were assembled based on historical records. Comparisons of categorical variables used Pearson's Chi-square and Fisher's exact test while continuous values were compared using the Wilcoxon signed-rank test. Survival comparisons were assessed using Kaplan-Meier statistics and Cox regressions.

**Results:** Seventy-one gliosarcoma patients were identified. Secondary gliosarcoma was not associated with worse survival when compared to recurrent primary gliosarcoma (median survival 9.8 [3.8 to 21.0] months vs. 7.6 [1.0 to 35.7], *p* = 0.7493). On multivariable analysis, receipt of temozolomide (HR = 0.02, 95% CI 0.001–0.21) and achievement of gross total resection (GTR; HR = 0.13, 95% CI 0.02–0.77) were independently prognostic for improved progression-free survival (PFS) while only receipt of temozolomide was independently associated with extended overall survival (OS) (HR = 0.03, 95% CI 0.001–0.89). In patients receiving surgical resection followed by radiotherapy and concomitant temozolomide, achievement of GTR was significantly associated with improved PFS (median 32.97 [7.1–79.6] months vs. 5.45 [1.8–26.3], *p* = 0.0092) and OS (median 56.73 months [7.8–104.5] vs. 14.83 [3.8 to 29.1], *p* = 0.0252).

**Conclusion:** Multimodal therapy is associated with improved survival in gliosarcoma. Even in patients receiving aggressive post-operative multimodal management, total surgical removal of macroscopic disease remains important for optimal outcomes.

## Introduction

Gliosarcomas are recognized by the World Health Organization (WHO) as a distinct subtype of glioblastoma. The age-adjusted incidence of gliosarcoma is 3.2 cases per 100,000 and comprises more than half of newly-diagnosed neuroepithelial tumors of the central nervous system (CNS) (1). Histologically, gliosarcomas can be distinguished by the biphasic presence of both glial and sarcomatous components (2–4). Molecular studies on gliosarcoma have suggested a monoclonal origin of these distinct cell populations prior to their divergent differentiation patterns based on the presence of shared somatic alterations, including that of tumor suppressors *TP53* and *PTEN* (5–7).

Evaluation of gliosarcoma response to therapy is limited due to its rarity. As most chemotherapeutic agents demonstrate limited efficacy in CNS malignancies, gliosarcoma treatments generally parallel that of glioblastoma, consisting of maximal surgical resection, temozolomide (TMZ), and radiotherapy (8). Some studies demonstrate a possible benefit associated with TMZ and radiotherapy (9, 10); however, prospective evidence is sorely lacking (11–13). Although gross total resection (GTR) of glioblastoma has been associated with improved progression free survival (PFS) independent of adjuvant therapy, this has not yet been demonstrated in gliosarcoma (11).

For this study, we have compiled our institutional series of histologically confirmed gliosarcoma cases to investigate the additive effect of GTR compared to near total and subtotal resection in patients receiving post-operative TMZ.

## Methods

### Study Cohort

The study cohort consisted of patients with histologically confirmed gliosarcoma diagnosed between 1999 and 2019 at a single center. Histological classification was based on the presence of both GFAP-positive glial and reticulin-positive sarcomatous cells upon immunohistochemical staining. Patients were queried using STARR [Stanford Research Repository [formerly known as STRIDE]], a prospectively-collated resource allowing for comprehensive and systematic identification of historical cohorts (14). Retrospective review of clinical records was conducted to aggregate demographic, treatment, and disease-specific characteristics for subsequent analyses. Patients were classified as either primary or secondary gliosarcoma based on evidence of a prior high-grade glial tumor. This retrospective study was approved by the Stanford University Institutional Review Board prior to data collection and analysis.

### Data Assembly

Internal and obtainable external records were assessed for demographic, treatment, and tumor characteristics. Clinical variables acquired included age, sex, race, symptoms at initial diagnosis, Karnofsky performance score (KPS), tumor molecular characteristics (e.g., *IDH1* mutation status, *MGMT* promotor methylation, *EGFR* alterations, *TP53* expression), lesion location, laterality, and pre-surgical size. Tumor localization and appearance at initial presentation was assembled using magnetic resonance (MR) or computerized tomography (CT) studies at diagnosis. Percentages were calculated based on patients for whom characteristics were discernable (excluding unknowns). Patients with at least one clinical or radiological follow-up after the initial surgical admission constitute the “longitudinal analysis cohort” (Supplementary Table 1). As this study was intended to survey the clinicopathological presentation of gliosarcoma as well as long-term clinical outcomes, patients for whom follow-up was not available were also assembled (Table 1).

**Table 1:** Patient characteristics (Full cohort).

	**Combined**	**Primary GS**	**Secondary GS**
**Baseline demographics**	71 patients^†^	53 patients	15 patients
Year of diagnosis (median; range)	2011 (1999–2019)	2011 (2000–2019)	2011 (2000–2019)
Median age at diagnosis (range)	61.9 (2–88)	63.9 (9–88)	58.9 (2–72)
<50	21.6%	13.5%	42.9%
≥50– ≤ 70	56.9%	59.5%	50.0%
>70	21.6%	27.0%	7.1%
Median pre-operative KPS (range)	80 (40–100)	70 (0–100)	80 (70–80)
Median age of diagnosis of primary tumor	-	-	58 (2–70)
Median time to transformation (mo.)	-	-	11 (0.4–134)
Gender			
Male	60.3%	62.3%	53.3%
Female	39.7%	37.7%	46.7%
**Disease characteristics**			
Location			
Multilobar	18.3%	18.9%	20.0%
Frontal	16.9%	15.1%	26.7%
Temporal	38.0%	35.8%	53.3%
Parietal	15.5%	18.9%	0
Occipital	1.4%	1.9%	0
Other	4.2%	3.8%	0
Unknown	5.6%	5.7%	0
Laterality			
Unilateral	94.4%	94.3%	100.0%
Bilateral	1.4%	1.9%	0
Unknown	4.2%	3.8%	0
History of RT			
Yes	23.1%	4.0%	86.7%
No	76.9%	96.0%	13.3%
History of systemic therapy			
Yes	20.6%	2.1%	80.0%
No	79.4%	97.9%	20.0%
**Treatment characteristics**			
Surgery			
Yes	88.9%	90.4%	83.3%
No	11.1%	9.6%	16.7%
Extent of resection			
GTR	38.6%	38.9%	37.5%
STR/NTR	47.7%	50.0%	37.5%
Biopsy	13.6%	11.1%	25.0%
Adjuvant systemic therapy			
Yes	76.2%	76.7%	72.7%
No	23.8%	23.3%	27.3%
Temozolomide			
Yes	60.0%	68.0%	33.3%
No	40.0%	32.0%	66.7%
Bevacizumab			
Yes	34.2%	25.0%	66.7%
No	65.8%	75.0%	33.3%
Adjuvant RT			
Yes	67.4%	74.2%	45.5%
No	32.6%	25.8%	54.5
Multiple surgeries			
Yes	18.3%	18.9%	20.0%
No	81.7%	81.1%	80.0%

† *Includes 3 patients who were not identifiable as either primary or secondary gliosarcoma (GS)*.

Treatment-specific data collected include that of surgery (timing, extent of resection, subsequent re-resections, and concurrence with additional therapies), radiotherapy (RT) (timing, modality, dose, and fractionation), and systemic therapy (timing, therapeutic agent, and dose). Patients with “near total resection” were classified as receiving “subtotal resection” as both reflect residual macroscopic tumor following resection. Extent of resection was extracted from a combination of operative notes and postoperative radiology assessments. Disease progression was defined as the date of radiographic recurrence necessitating either surgical intervention or initiation of salvage therapy. Transformation time was determined based on the time elapsed between radiographic or histologic diagnosis of the primary glial tumor and first visualization of the lesion histopathologically classified as gliosarcoma. Outcomes were progression-free survival and overall survival, as assessed during clinical visits and imaging studies. Survival was assessed from the date of radiographic imaging immediately preceding surgical resection, on which tumor size was assessed.

### Statistical Analysis

Frequency differences in baseline categorical variables were interrogated using Pearson's chi-squared and Fisher's exact testing. Wilcoxon rank-sum statistics was used to evaluate differences in continuous variables. Differences in time to either progression or death were evaluated using Cox proportional hazards regression. To ensure reduced dimensionality of the multivariable analysis (necessary given the limited cohort size), only variables with a *p* < 0.1, a heuristic defined *a priori*, were included in the multivariable model. Survival curve differences were evaluated using the Mantel-Cox method and hazard rates were determined based on the Cox proportional hazards assumption. All statistical tests were two-sided and evaluated based on an α of 0.05. Analyses and graphical representations were performed using the R software package (version 3.4.3, The R Foundation for Statistical Computing) and GraphPad Prism 8 (GraphPad Software; San Diego, CA).

## Results

### Cohort Characteristics

**Figure 1:**
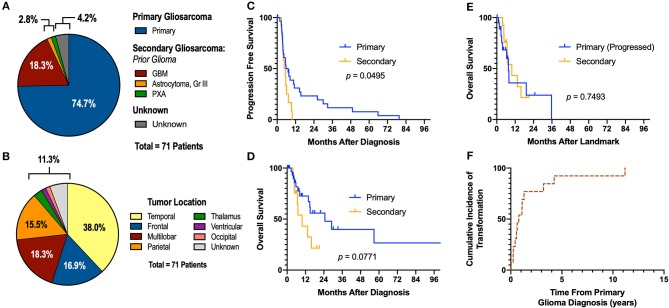
Descriptive Statistics of Gliosarcoma Cohort. **(A)** Etiology of gliosarcoma and **(B)** tumor location. **(C,D)** Primary gliosarcoma is associated with extended PFS compared to secondary gliosarcoma when measured from time of diagnosis. **(E)** Secondary gliosarcoma and recurrent primary gliosarcoma demonstrate similar survival. **(F)** Transformation to secondary gliosarcoma happens most frequently within the first 2 years of primary tumor diagnosis. GBM, glioblastoma multiforme; PXA, pleomorphic xanthoastrocytoma. One patient demonstrated glioblastoma at time of biopsy; examination of the resected lesion 0.4 months following the diagnostic biopsy demonstrated sarcomatous components not present at time of biopsy.

A total of 71 gliosarcoma patients with histologically-proven disease diagnosed between 1999 and 2019 were identified (Table 1). The majority of patients presented with primary gliosarcoma with no prior history of high-grade glial malignancy (77.9%, *n* = 53) while a subset of patients demonstrated gliosarcoma transformed from a prior high-grade glioma (22.1%, *n* = 15), most frequently glioblastoma (86.7% of secondary gliosarcoma, *n* = 13) (Figure 1A). Anatomical location was generally confined to a single lobe, primarily temporal (38.0%, *n* = 27) and frontal (16.9%, *n* = 12). However, a subset of patients demonstrated multilobar disease (18.3%, *n* = 13). Other tumor locations include parietal lobe (15.5%, *n* = 11), thalamus (2.8%, *n* = 2), ventricular (1.4%, *n* = 1), and occipital (1.4%, *n* = 1) (Figure 1B). Four patients had unknown tumor locations (due to unavailable clinical follow-up and imaging studies). One patient had a bilateral lesion (1.4%). In patients with records detailing neurological symptoms at presentation (*n* = 51), the most frequent symptoms were headache (*n* = 25, 49.0%), neuromuscular deficits (*n* = 18, 35.3%), and altered mental status (*n* = 13, 25.5%). Seizures (*n* = 10, 19.6%), nausea/vomiting (*n* = 7, 13.7%), and aphasia (*n* = 6, 11.8%) were also commonly observed at initial presentation. Median pre-operative Karnofsky performance score was 70 (range 40–100). None of the patients had metastatic disease at time of diagnosis.

### Treatment Patterns

All treatment and survival assessments were conducted on patients in the longitudinal analysis cohort (*n* = 46, Supplementary Table 1). The majority of the patients received surgery with at least one form of adjuvant therapy. Of the patients in the longitudinal analysis cohort, 43 (93.5%, 31 out of 33 primary gliosarcoma, 12 out of 13 secondary gliosarcoma) received surgery while 3 (6.5%) received biopsy only; operative data was not available for two patients (pathology consults without operative details).

Most primary gliosarcoma patients received radiotherapy following initial tumor resection (78.6%). All but one patient received fractionated external beam radiotherapy (EBRT); one patient received whole brain radiotherapy. Two patients received hypofractionated radiation (40 Gy in 15 fractions) while the remaining patients received 60 Gy in 30 fractions. Of the 6 patients not receiving post-operative radiotherapy, two were over the age of 80. Systemic therapy was administered in 84.0% of patients (excluding five patients in whom receipt of systemic therapy was unclear). Excluding one patient with unmethylated *MGMT* disease, all primary gliosarcoma patients diagnosed after 2005 received post-operative radiotherapy and concurrent temozolomide. Nine patients received re-resections following tumor progression (29.0%), with two patients also receiving stereotactic radiosurgery (SRS). Additional salvage therapies included bevacizumab (*n* = 5), TMZ (*n* = 2), lomustine (*n* = 3), carboplatin (*n* = 1), and tumor-treating fields (*n* = 1).

Of the patients with secondary gliosarcoma, only five patients received post-operative radiotherapy, though 11 patients had received radiation to the primary glial malignancy prior to transformation. Specific details on radiotherapy modality and course were not available for one patient. Of the remaining four secondary gliosarcomas treated with post-operative radiotherapy, two patients received fractionated EBRT (60 Gy in 30 fractions) while two received SRS (16 Gy single fraction). In lieu of post-operative radiotherapy, four patients received either TMZ or bevacizumab alone. Additional therapies following disease progression in secondary gliosarcoma patients included re-resections (*n* = 2), bevacizumab (*n* = 2), lomustine (*n* = 2), nivolumab (*n* = 1), carmustine (*n* = 1), tipifarnib (*n* = 1), and tumor-treating fields (*n* = 1).

During the course of treatment, 21 (45.7%) patients reported treatment-related adverse effects (Table 2). Adverse treatment-effects included hematological (6.5%, *n* = 3), thrombo-embolic (4.3%, *n* = 2), rash (4.3%, *n* = 2), infection (6.5%, *n* = 3), neuromuscular (6.5%, *n* = 3), steroid-induced (10.9%, *n* = 5), altered mental status (4.3%, *n* = 2), and radiation necrosis (2.8%, *n* = 1).

**Table 2:** Treatment-related adverse effects.

	**Total patients reporting**	**%**
Any adverse effect	21	45.7
Hematological	3	6.5
Thrombo-embolic	2	4.3
Rash	2	4.3
Infection	3	6.5
Neuromuscular	3	6.5
Sensory	2	4.3
Motor	1	2.2
Steroid-induced	5	10.9
Altered mental status	2	4.3
Radiation necrosis	1	2.8

### Factors Associated With Survival

In patients with longitudinal imaging follow-up after diagnostic biopsy or tumor resection (*n* = 46), median progression-free survival (PFS) was 5.55 months and median overall survival was 15.07 months. When measuring from first histological diagnosis of gliosarcoma, primary gliosarcoma patients demonstrated improved progression-free survival compared to those with secondary gliosarcoma (median PFS 6.45 [1.8–79.6] months vs. 5.00 [2.8–9.8], *p* = 0.0495, Figure 1C). Overall survival (OS) was also longer but failed to reach statistical significance (median OS 24.70 [range 2.6–104.5] months vs. 9.80 [3.8–21.0], *p* = 0.0771, Figure 1D). However, when comparing secondary gliosarcoma and progressive primary gliosarcoma landmarked at time of transformation or progression, respectively, no difference in overall survival was observed (median OS 9.8 [3.8–21.0] months vs. 7.6 [1.0–35.7], *p* = 0.7493, Figure 1E). In the patients with secondary gliosarcoma and known diagnosis date of the preceding high-grade glioma, median transformation time was 9.44 months with 53.8% (*n* = 7 out of 13) transforming within 1 year (Figure 1F).

Patient age, gender, tumor location, tumor size, *MGMT* promotor methylation status, and extent of resection, along with receipt of radiotherapy, TMZ, and bevacizumab, were included in univariable analyses. Only primary gliosarcoma patients were included as prior tumor histology and past cancer treatment are likely to affect outcomes in secondary gliosarcoma. On univariable analysis, patients receiving GTR experienced improved progression-free survival [hazard ratio [HR] = 0.34, 95% confidence interval [CI] 0.13–0.89]. Overall survival also improved but did not reach statistical significance (HR = 0.30, 95% CI 0.08–1.15). Use of radiotherapy was also associated with prolonged progression-free survival (HR = 0.25, 95% CI 0.08–0.84) but not overall survival (HR = 0.22, 95% CI 0.02–2.33). *MGMT* promotor methylation was associated with improved overall survival (HR = 0.08, 95% CI = 0.01–0.91) but not progression-free survival (HR = 0.27, 95% CI 0.07–1.04). Receipt of TMZ portended both improved progression-free survival (HR = 0.11, 95% CI 0.03–0.44) and overall survival (HR = 0. 05, 95% CI 0.003–0.88). Bevacizumab treatment was not associated with differences in either progression-free survival (HR = 1.56, 95% CI 0.61–4.00) or overall survival (HR = 1.77, 95% CI 0.47–6.67).

**Figure 2:**
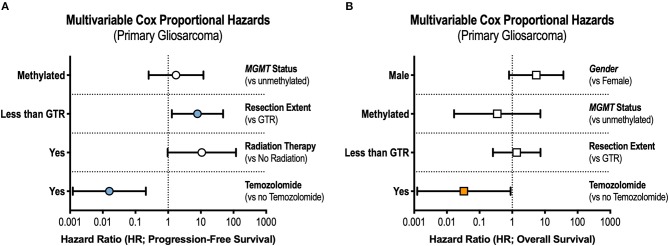
Prognostic Factors in Gliosarcoma. Multivariable Cox regression interrogating covariates independently associated with **(A)** progression-free survival and **(B)** overall survival. GTR, gross total resection.

In the multivariable Cox model, receipt of GTR (adjusted hazard ratio [aHR] = 0.13, 95% CI 0.02–0.77) and TMZ (aHR = 0.02, 95% CI 0.001–0.21) were independently associated with prolonged progression-free survival (Figure 2A). Only treatment with TMZ was associated with improvements in overall survival in the adjusted analysis (aHR = 0.03, 95% CI 0.001–0.89, Figure 2B).

### Subset Analyses in Gliosarcoma Treated With Temozolomide

All primary gliosarcoma patients treated with concurrent and adjuvant TMZ received post-operative radiotherapy (*n* = 19). Of these, *MGMT* status was available in 11 patients (57.9%). In this subset, the majority of patients demonstrated *MGMT* silencing (*n* = 8, 72.7%). Despite clinically significant improvements in median progression-free survival (median 14.57 [1.8–79.6] months vs. 8.433 [3.5–10.7], *p* = 0.2316) and overall survival (median 101.6 [4.5–104.5] months vs. 14.38 [9.2–14.8], *p* = 0.3558) in patients with *MGMT* silencing, neither result reached statistical significance.

**Figure 3:**
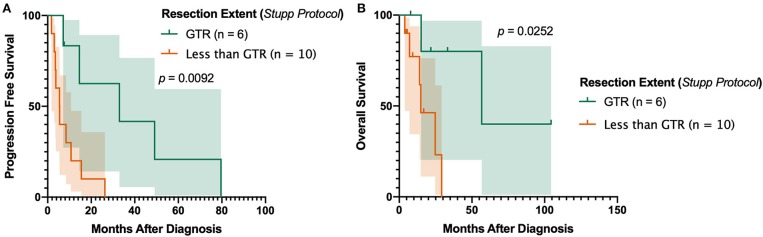
Impact of Resection Extent in Patients Receiving Stupp Protocol. Achievement of gross total resection is associated with improved **(A)** progression-free survival and **(B)** overall survival in patients uniformly receiving surgery, post-operative radiotherapy, and concomitant and adjuvant temozolomide. GTR, gross total resection.

In the subset of patients receiving post-operative TMZ and radiotherapy for primary gliosarcoma, gross total resection (GTR) was associated with improved outcomes. Patients receiving GTR had significantly prolonged progression-free survival compared to those with macroscopic residual tumor (either subtotal resection [STR] or near total resection [NTR]) (*p* = 0.0224). No patients received biopsy without subsequent definitive tumor resection. Overall survival was also substantially increased despite not reaching statistical significance (*p* = 0.0818). Sixteen (84.2%) primary gliosarcoma patients receiving TMZ and radiation received concurrent chemoradiation and adjuvant chemotherapy in accordance with the Stupp protocol^37^. In this subset (*n* = 16), GTR improved both progression-free survival (median PFS 32.97 [7.1–79.6] months vs. 5.45 [1.8–26.3], *p* = 0.0092, Figure 3A) and overall survival (median OS 56.73 [7.8 to 104.5] months vs. 14.83 [3.8–29.1], *p* = 0.0252, Figure 3B) compared to patients receiving STR or NTR.

## Discussion

In the historical series of gliosarcoma diagnosed or treated at our institution, we identified the importance of maximal surgical resection and temozolomide (TMZ) treatment in achieving prolonged progression-free and overall survival. Importantly, we demonstrated improved survival among patients receiving gross total resection (GTR) compared to subtotal (STR) or near total resection (NTR) in gliosarcomas receiving modern medical and radiotherapeutic management, emphasizing the importance of complete macroscopic tumor resection, even in the context of aggressive multimodal adjuvant therapy.

Gliosarcoma is often diagnosed in the sixth decade, has a male predominance, and a predilection for the temporal lobe, all evident in our series (9, 15, 16). Consistent with previous reports (17, 18), secondary gliosarcoma accounted for 22% of all histologically-confirmed gliosarcoma cases in our study. Among gliosarcoma patients, the estimated median overall survival landmarked from time of gliosarcoma diagnosis was 15 months, though this estimate was much higher when only primary gliosarcoma patients were included (24.7 months). Previous retrospective studies on primary gliosarcoma report estimates ranging from 5.7 to 16.7 months (10, 19–24). While our estimate is higher than what is typically reported, it aligns with a previous published estimate from a similar large academic center where patients were also frequently enrolled in clinical trials (25). In addition, our cohort consists of more recently treated patients diagnosed through 2019, which may reflect the widespread adoption of concurrent and adjuvant TMZ plus radiotherapy demonstrated to enhance survival in glioblastoma, possibly leading to improved outcomes (26).

Some reports demonstrate secondary gliosarcoma carrying worse prognoses (25), while others demonstrate shorter survival among primary disease (17). Though our cohort supports the notion that secondary gliosarcoma confers poorer prognoses when accruing time from gliosarcoma diagnosis (progression-free [median 5.0 months vs. 6.5] and overall survival [median 9.8 months vs. 24.7]), comparison of progressive primary gliosarcoma to secondary gliosarcoma, landmarking at time of progression or transformation, demonstrates no difference in overall survival (median 7.6 months vs. 9.8). Therefore, it is possible that histological transformation, while biologically distinct from progression of primary gliosarcoma, might yield similar clinical outcomes. However, conflicting reports among the literature warrant additional studies to better understand the prognostic significance of a prior glioma preceding gliosarcoma diagnosis.

Gross total resection has been widely shown to be prognostic for improved outcomes compared to subtotal resection in glioblastoma (27, 28); however, its role in extending survival in gliosarcoma, particularly in the TMZ era, is less understood. Prior database studies in the Surveillance, Epidemiology, and End Results (SEER) Program (23, 29) and the National Cancer Database (NCDB) (30, 31) have suggested a potential benefit of achieving GTR. Despite these findings, neither SEER nor NCDB contain data on the specific chemotherapeutic agents administered making it impossible to deconvolute granular details on post-surgical management. Primary and secondary gliosarcoma are also difficult to distinguish using either SEER or NCDB, limiting the interpretability of large-scale database studies on gliosarcoma. Smith et al. recently published a series of gliosarcoma in which they demonstrated that, compared to biopsy, achievement of GTR improves survival (12). No independent association with survival was found when comparing patients receiving GTR to those receiving surgical resection less than GTR, however. In a series of gliosarcoma patients uniformly treated according to the Stupp protocol (26), GTR was not significantly associated with survival compared to STR (11). In contrast, our findings suggest any discernable macroscopic residual disease (either STR or NTR) is associated with worse overall and progression-free survival compared to patient receiving GTR (Figure 3). Despite the inherent biases of retrospective studies, these results suggest complete removal of the tumor is necessary for maximal survival, even in the context of multimodal post-operative management.

Prior to 2005, external beam radiation (EBRT) alone was the most common post-operative treatment regimen. However, all but one primary gliosarcoma patients diagnosed post-2005 received post-operative combined chemoradiation with TMZ, reflecting the widespread adoption of the Stupp protocol. While TMZ is commonly used to treat high-grade glial tumors, such as glioblastoma, it remains unclear how the presence of a non-glial tumor component affects response to TMZ. Additionally, while clinical management of newly diagnosed primary gliosarcoma in the TMZ era has modeled that of glioblastoma, there is no consensus on how to treat secondary gliosarcoma or primary gliosarcoma following disease progression and efforts to characterize responses to salvage therapies are sparse.

While prior studies have suggested a higher predilection of gliosarcoma to present with extracranial metastases, we identified only a single patient with histologically confirmed metastatic gliosarcoma. This patient received an NTR of his primary right temporal gliosarcoma, subsequently receiving post-operative fractionated intensity-modulated radiotherapy (60 Gy in 30 fractions) and TMZ. Upon progression, repeat resection of the lesion yielded GTR and the patient was continued on TMZ post-operatively. During a subsequent admission for treatment of a surgical site infection, bilateral pulmonary masses were identified by chest x-ray, and subsequent CT-guided biopsy confirmed metastatic disease. Interestingly, the biopsied metastatic lesion demonstrated dominance of the sarcomatous component of the primary lesion, consistent with prior literature suggesting preferential spread of the sarcomatous fraction (32, 33). Over the next 5 months, the patient developed widespread metastatic disease, including osseous metastases in both the ribs, thoracic spine, and lumbar spine, and the patient expired soon after. Three other patients demonstrated radiographically apparent extracranial lesions, but pathology was not available to confirm metastatic disease vs. a synchronous extracranial primary tumor.

Two cases of pediatric gliosarcoma were included in this series. The first was a case of secondary gliosarcoma of the left frontal lobe transforming from a primary anaplastic pleomorphic xanthoastrocytoma (PXA). While the vast majority of secondary gliosarcomas arise from a prior glioblastoma, rarely, gliosarcoma has been reported to arise from malignant transformation of other high grade glial tumors (34). Only a handful of anaplastic PXA cases have been reported in literature (35–38), and the transformation of PXA to secondary gliosarcoma has never been documented before in the pediatric population (39). After subtotal resection of the left frontal malignant PXA, this patient was enrolled in a clinical trial for O(6)-benzylguanine and carmustine, which was discontinued following disease progression. Pathology obtained following debulking of the recurrent lesion was consistent with gliosarcoma, and the patient then received palliative radiation therapy to extra-axial growths including bony disease invasion involving the superior left orbital rim and nodular lesions along the lower jaw and left ear (presumably gliosarcoma metastasis but histology was unavailable for confirmation). The patient passed approximately 4 months later. In addition, three patients demonstrated gliosarcoma with primitive neuroectodermal tumor (PNET) components; the presence of PNET-components is not well-documented nor is its prognostic significance understood (40, 41).

Limitations of this study include small cohort size, heterogeneity in management and follow-up, and its retrospective nature. Gliosarcoma is an extremely rare histological variant of glioblastoma and, despite this cohort representing one of the larger institutional series assembled to date, the limited cohort size nonetheless restricts potential subset analyses and could hamper our ability to adequately adjust for confounding covariates. Furthermore, management of gliosarcoma is heterogeneous, particularly in patients diagnosed prior to the TMZ era (pre-2005). In such patients, conducting adequately powered analyses of therapeutic efficacy is challenging. As many of the patients were consults referred to our institution from local care centers, diverse care settings also contributed to treatment and follow-up heterogeneity. The retrospective nature of this study must also be emphasized, as despite best efforts to adjust for selection bias through multivariable analyses, biases remain a potential issue and additional randomized, prospective studies are required to definitively demonstrate causative relationships posited in this study.

## Conclusion

Gliosarcoma is a distinct histological subtype of glioblastoma responsive to alkylating DNA damage caused by temozolomide administered concurrent with and adjuvant to radiotherapy (per Stupp protocol). Despite the retrospective nature of this study and the limited cohort size, our results indicate gross total resection may be important for achieving best outcomes, even in the context of extensive post-operative management through systemic and radiotherapeutic measures. Additional prospective studies are warranted to further establish best practices for clinical management of gliosarcoma.

## Data Availability Statement

The datasets generated for this study are available on request to the corresponding author.

## Ethics Statement

This retrospective study was approved by the Stanford University Institutional Review Board prior to data collection and analysis. Written informed consent was obtained from all individual participants included in the study.

## Author Contributions

MJ, EL, SN, and GL conceived the project. MJ and EL acquired requisite data, conducted statistical analyses, interpreted data, generated figures and tables, drafted the manuscript, and received final manuscript submission. SS, IG, RT, LR, SS, EP, SC, MH, and GL critically revised manuscript draft.

### Conflict of Interest

The authors declare that the research was conducted in the absence of any commercial or financial relationships that could be construed as a potential conflict of interest.
