# Supplementary material for: Evaluating Surgical Resection Extent and Adjuvant Therapy in the Management of Gliosarcoma
Source: Front Oncol. 2020 Mar 11;10:337. doi: 10.3389/fonc.2020.00337 (PMC7078164; doi:10.3389/fonc.2020.00337)
Supplement: Supplementary file 1 [file Table_1.docx]

| **SUPPLEMENTARY TABLE 1: Patient Characteristics (Longitudinal Cohort)** | | | |
| --- | --- | --- | --- |
|  | **Combined** | **Primary GS** | **Secondary GS** |
| **Baseline demographics** | 46 | 33 | 13 |
| Year of diagnosis (median; range) | 2011 (1999-2019) | 2010 (2000-2019) | 2015 (1999-2018) |
| Median age at diagnosis (range) | 62.3 (2-88) | 63.9 (38-88) | 56.3 (2-72) |
| <50 | 23.9% | 15.2% | 46.2% |
| ≥50-≤70 | 52.2% | 57.6% | 38.5% |
| >70 | 23.9% | 27.3% | 15.4% |
| Median pre-operative KPS (range) | 80 (70-100) | 85 (70-100) | 80 (70-80) |
| Median age of diagnosis of primary tumor | 61.6 (2-88) | 63.9 (38-88) | 55.5 (2-70) |
| Median time to transformation (mo.) | - | - | 9.4 (0.46-134) |
| Gender |  |  |  |
| Male | 56.5% | 57.6% | 53.8% |
| Female | 43.5% | 42.4% | 46 .2% |
| **Disease characteristics** |  |  |  |
| Location |  |  |  |
| Multilobar | 19.6% | 18.2% | 23.1% |
| Frontal | 17.4% | 15.2% | 23.1% |
| Temporal | 47.8% | 45.4% | 53.8% |
| Parietal | 10.9% | 15.2% | 0 |
| Occipital | 2.2% | 3.0% | 0 |
| Other | 2.2% | 3.0% | 0 |
| Unknown | 0 | 0 | 0 |
| Laterality |  |  |  |
| Unilateral | 97.8% | 97.0% | 100.0% |
| Bilateral | 0 | 0 | 0 |
| Unknown | 2.2% | 3.0% | 0 |
| History of RT |  |  |  |
| Yes | 26.1% | 3.0% | 84.6% |
| No | 73.9% | 97.0% | 15.4% |
| Unknown | 0 | 0 | 0 |
| History of systemic therapy |  |  |  |
| Yes | 26.1% | 3.0% | 84.6% |
| No | 69.6% | 90.9% | 15.4% |
| Unknown | 4.3% | 6.1% | 0 |
| **Treatment characteristics** |  |  |  |
| Surgery |  |  |  |
| Yes | 93.5% | 93.9% | 92.3% |
| No | 6.5% | 6.1% | 7.7% |
| Extent of resection |  |  |  |
| GTR | 42.1% | 41.9% | 42.9% |
| STR/NTR | 50.0% | 51.6% | 42.9% |
| Biopsy | 7.9% | 6.4% | 14.3% |
| Adjuvant systemic therapy |  |  |  |
| Yes | 82.9% | 84.0% | 80.0% |
| No | 17.1% | 16.0% | 20.0% |
| Temozolomide |  |  |  |
| Yes | 63.6% | 79.2% | 22.2% |
| No | 36.4% | 20.8% | 81.8% |
| Bevacizumab |  |  |  |
| Yes | 39.4% | 29.2% | 66.7% |
| No | 60.6% | 70.8% | 33.3% |
| Adjuvant RT |  |  |  |
| Yes | 69.2% | 78.6% | 45.4% |
| No | 30.8% | 21.4% | 54.6% |
| Multiple Surgeries |  |  |  |
| Yes | 28.3% | 30.3% | 23.1% |
| No | 71.7% | 69.7% | 76.9% |
